# A randomized controlled feasibility study to evaluate the effects of a goal-setting coaching intervention using feedback from an accelerometer on sedentary time in older people at risk of falls (SMART-MOVE): a study protocol

**DOI:** 10.1186/s40814-018-0366-5

**Published:** 2018-11-17

**Authors:** Kareeann Sok-Fun Khow, Joanne Dollard, Kathy Bray, Carla Smyth, Mellick Chehade, Olga Theou, Renuka Visvanathan

**Affiliations:** 1 National Health and Medical Research Council Centre for Research Excellence in Frailty and Healthy Ageing, Adelaide, South Australia Australia; 2 Adelaide Geriatrics Training and Research with Aged Care (G-TRAC Centre), Discipline of Medicine, Adelaide Medical School, University of Adelaide, 61 Silkes Road Paradise, Adelaide, South Australia SA 5075 Australia; 3 Aged and Extended Care Services, The Queen Elizabeth Hospital, Central Adelaide Local Health Network, SA Health, Adelaide, South Australia Australia; 4 Centre for Orthopaedic Trauma and Research, Royal Adelaide Hospital, The University of Adelaide, Adelaide, South Australia Australia; 5 Geriatric Medicine, Dalhousie University, Halifax, Canada

**Keywords:** Accelerometer, Falls, Health coaching, Older people, Sedentary behaviour

## Abstract

**Background:**

Sedentary behaviour and falls are important interrelated health issues in older people. One in three people aged 65 years and above fall at least once a year and sedentary behaviour has been identified as one of the risk factors for falls. Studies have shown that the duration of sedentary time increases with age. These dual problems need to be addressed effectively as the ageing population grows. Accelerometers enable accurate measurement of sedentary time. This study aims to establish the feasibility and effect of an individualized goal-setting health coaching intervention using feedback initially from an accelerometer and then pedometer over a period of 12 weeks (intervention) compared with providing a one-off advice through a brochure (control), on sedentary time in older people with a recent fall or at risk of one.

**Methods:**

A single-blinded randomized controlled feasibility trial involving 80 community-dwelling people aged 65 years and above will be conducted with 40 randomized to the intervention and another 40 to control. Primary outcomes will be the feasibility of the intervention and change in total sedentary time at 12 and 24 weeks. Secondary outcomes include a change in fear of falling based on the falls efficacy scale, gait speed, self-reported sedentary time, the proportion of fallers and number of falls. Four focus groups (two from each arm) will be conducted at the end of the study to evaluate the feasibility and effectiveness of this intervention. Feasibility findings will be primarily descriptive. Mean group differences will be examined using independent samples *t* test for normally distributed data and nonparametric tests (Kruskal-Wallis and Mann-Whitney *U*) for non-normally distributed data. Differences in frequency of variables will be compared using chi-square test. Analysis of variance (ANOVA) will be used to test the post-intervention difference between the two groups at 12 and 24 weeks.

**Discussion:**

The trial will address a key gap in evidence about sedentary behaviour and falls amongst older people and will evaluate the feasibility of an intervention that could be implemented within the primary health care settings.

**Trial registration:**

Australian New Zealand Clinical Trials Registry 12617001186347, Registered 11 August 2017.

## Background

Falls are common among older people. It is estimated that about 30% of people aged 65 years or older fall at least once a year [1]. In Australia, this approximates to one million people annually [2]. Falls are a major contributor to injuries, disability and premature death, substantially impacting on the health and independence of older people [3]. The economic costs of falls are huge, with an annual direct medical cost of $30 billion in the USA [4]. Therefore, the prevention of falls among older people must be addressed urgently because the burden of this problem will only increase if no intervention is undertaken.

Falls are associated with many risk factors, and sedentary behaviour is one of them [5]. Sedentary behaviour refers to low-energy expending behaviour undertaken while sitting or lying down when awake [6]. Older people have been found to be sedentary more than 70% of their time, i.e., 8–10 h of their waking day, and this increases linearly with age [7]. A meta-analysis of eight observational studies has reported that sedentary behaviour was associated with an increased risk of falls [8]. Lower extremity muscle weakness and balance impairment may be mediating factors between sedentary behaviour and falls [8].

Furthermore, there is also concern that sedentary behaviour increases after older people have had falls [9]. Fear of falling and slow gait speed are associated with an increased risk of falls. Low falls efficacy scale (FES), a measure of fear of falling, is associated with an increased risk of subsequent falls, a decline in activities of daily living and reduced quality of life [3]. The association between gait speed and falls is non-linear, with a higher risk of falls outdoor among faster speed walkers, and falls indoor among slow-speed walkers [10].

There is a growing body of evidence that has shown multifactorial interventions including exercise programs especially those that improve balance and strength training, gait training, medication reviews, home risk modifications and education are effective to prevent falls in community-dwelling older people [11]. Many intervention studies aimed at increasing physical activity have been used to reduce falls [12]. However, no study has evaluated whether the reduction in sedentary behaviour can influence falls risk and only a few have examined effective ways to reduce sedentary behaviour among older people [13–16]. Moreover, standardized supervised exercise interventions do not necessarily reduce sedentary behaviour in the older population [17]. Increasing physical activity does not always result in reduced sedentary time. Therefore, reducing sedentary behaviour is a distinct domain and has to be addressed separately from increasing physical activity [18].

To date, there is only one randomized controlled trial comparing reducing sedentary behaviour (“Sit Less” group) and increasing moderate to vigorous physical activity (MVPA) (“Get Active” group) among 38 community-dwelling older people aged > 60 years, for a period of 12 weeks [19]. The goal for the “Sit Less” group was to reduce sedentary time by 60 min each day while the “Get Active” group was to reach 150 min of MVPA each week. Both groups received a combination of individual, face-to-face and phone consultations with an exercise physiologist. Sedentary time did not change in either group but the “Sit Less” group improved their short physical performance battery (SPPB) score by 0.5 ± 0.3.

Accelerometers are devices used to measure the acceleration of movement that can be categorized into different intensities of activity. One such accelerometer, ActivPAL, is useful for measuring free-living sedentary behaviour [20]. Self-monitoring technologies such as pedometers are increasingly used as motivational tools to increase physical activity. Pedometers are simple and inexpensive body-worn movement sensors. Pedometers have been found to be associated with improvement in physical activity [21]. Several studies have demonstrated that pedometers can successfully increase physical activity in older people [22–24]. Therefore, the use of pedometers may have a role in reducing sedentary behaviour by providing immediate and objective feedback.

Health coaching is the practice of health education and health promotion within a coaching context, to enhance the well-being of individuals and to facilitate the achievement of their health-related goals [25]. In health coaching, a coach helps the participant to achieve their health-related goals by facilitating the learning process. Health coaching has been found to be an effective method to support behaviour change amongst older people. Health coaching based on behaviour change theories such as self-determination theory (SDT) has a strong evidence of efficacy [26]. SDT targets perceptions of autonomy, competence and relatedness to increase autonomous motivation, that is volitional and internal rather than external [27]. Using health coaching to reduce sedentary duration in older people is still a new approach. The Coventry, Aberdeen and London—Refined (CALO-RE) taxonomy is a tool to specify the content of behavioural change interventions [28]. Nine domains in this taxonomy are useful in designing health coaching interventions. Each domain describes a technique that is used in influencing an aspect of participants’ behaviour. These domains include shaping knowledge about health consequences, goal-setting (behaviour), goal-setting (outcome), feedback on behaviour, action planning, barrier identification or problem-solving, use of follow-up prompts, review of goals and relapse prevention.

Four small pre- and post-intervention studies in community-dwelling older people have been conducted using health coaching to reduce sedentary behaviour [13–16]. These studies reported reductions in objectively measured sedentary time ranging between 24 and 51 min per day. However, the main limitation with these studies was a short duration of intervention (between 1 and 14 days) [13, 15, 16]. To date, no studies on reducing sedentary behaviour have targeted older people with a history of falls or falls risk. Therefore, an individualized goal-setting intervention has been developed to reduce sedentary behaviour by using health coaching based on initial accelerometer and then pedometer over 12 weeks.

## Aims

The primary aim of this trial is to evaluate the feasibility and effect of a goal-setting health coaching intervention based on initial accelerometer and then pedometer feedback on total sedentary time for 12 weeks compared to one-off advice through a brochure in reducing sedentary behaviour in older people at high risk of falling according to the STEADI criteria (had a fall in last 12 months, feel unsteady when walking or standing, and/or worry about falling) [29].

The secondary aims are to compare the change in sedentary time based on the self-reported Measure of Older Adults' Sedentary Time (MOST) questionnaire [30], FES [31], change in gait speed, proportion of fallers and numbers of falls.

Evaluation of effects at 24 weeks will also occur to determine if the changes seen with the intervention compared to control is sustained at 24 weeks.

It is hypothesized that the intervention will be feasible with a 40% recruitment rate and an 80% retention rate will be achieved at 6 months. For adherence and acceptability, it is hypothesized that goal attainment scale of 0 (indicating goal achieved) for at least three goals will be achieved in 70% of participants (intervention group) and the intervention is acceptable in 70% of the participants. A further hypothesis is that the intervention is effective in reducing sedentary time and the benefits sustained at 24 weeks.

## Methods/design

This trial has been designed in accordance with the CONsolidated Standards of Reporting Trials (CONSORT) statement [32, 33] and is reported according to the Standard Protocol Items: Recommendations for Interventional Trials (SPIRIT) statement [34] and with reference to the Template for Intervention Description and Replication (TIDieR) checklist (see Table 1) [35].

**Table 1:** Intervention description using the Template for Intervention Description and Replication (TiDieR) checklist

1. Brief name	SMART-MOVE Specific, Measurable, Attainable, Relevant and Time-bound (SMART) Goal-setting coaching and accelerometer feedback to reduce older people’s sedentary time (MOVE)
2. Why	Excess time in sedentary behaviour is a prevalent health risk in the older population. Even amongst those who achieved the recommended requirement for physical activity, sedentary behaviour is considered detrimental and increasingly recognized as a health risk independent of physical activity. It is associated with adverse outcomes, such as falls, which is in turn a risk factor for fragility fractures. There is an urgent need to address this issue as the number of older people is expected to increase due to population ageing. Few studies have evaluated interventions to increase physical activity levels in older people who are at risk of falls. The coaching intervention is based on self-determination theory on modification of behaviour.
3. What—materials	Participants will receive: • The ‘Choose Health: Be Active’ booklet developed by the Australian Government in collaboration with Department of Veterans’ Affairs and Department of Health and Aging to help older Australians achieve sufficient physical activity for good health as they age. • An accelerometer to record sedentary behaviour for a week (at week 1, 12 and 24). • A SMART goal-setting booklet will be given to participants in the intervention group. • A pedometer to measure daily step counts.
4. What—procedures	Face-to-face coaching with goal setting will occur after accelerometer reading is available at the start of the study and then at week 6. Subsequently, telephone interviews will occur fortnightly on four occasions (week 2, 4, 8 and 10) to identify barriers and assist participants to achieve their physical activity goals.
5. Who provided	Two researchers with professional backgrounds in medicine and nursing will deliver the intervention.
6. How	The intervention will be tailored to suit the participant daily activities. SMART (specific, measurable, attainable, relevant, time-bound) goals will be set. At the first face-to-face meeting, participants will set three goals to reduce sedentary behaviour. One goal will be incrementally introduced every 2 weeks so that by week 6, the participant will be working on three goals. At the second face-to-face meeting, the participant will set another three goals that will be added incrementally every fortnight. Participants in the intervention arm will be provided with information about their duration of time spent upright (accelerometer) and total number of steps (pedometer) taken each day. Goals are set to increase their time spent upright and total number of steps taken. Participants will calculate the mean daily steps over 7 days and increase by 200 steps from the mean per week as a goal. They will also be encouraged to attend falls prevention classes if they have not participated in one in the preceding 12 months.
7. Where	The intervention will be delivered to community-dwelling older people who had at least one fall in the last 12 months or are at risk of one. It will be delivered at Adelaide G-TRAC Centre or The Queen Elizabeth Hospital.
8. When and how much	The face-to-face assessment, goal setting and health-coaching will occur at the beginning of the intervention period and will last approximately 2 h. An accelerometer will be worn for one week before this face-to-face coaching. Phone coaching will occur for up to 15 min fortnightly when there is no face-to-face coaching. At week 6, a second face-to-face coaching will be conducted where goals will be reviewed and additional goals set.
9. Tailoring	The recommended physical activity plan will be tailored to individual needs based on participants’ goals, baseline levels, preferences and physical ability.

### Ethics

This study has been approved by The Queen Elizabeth Hospital (TQEH) Human Research Ethics Committee (Reference number: HREC/17/TQEH/58).

### Study design

**Fig. 1:**
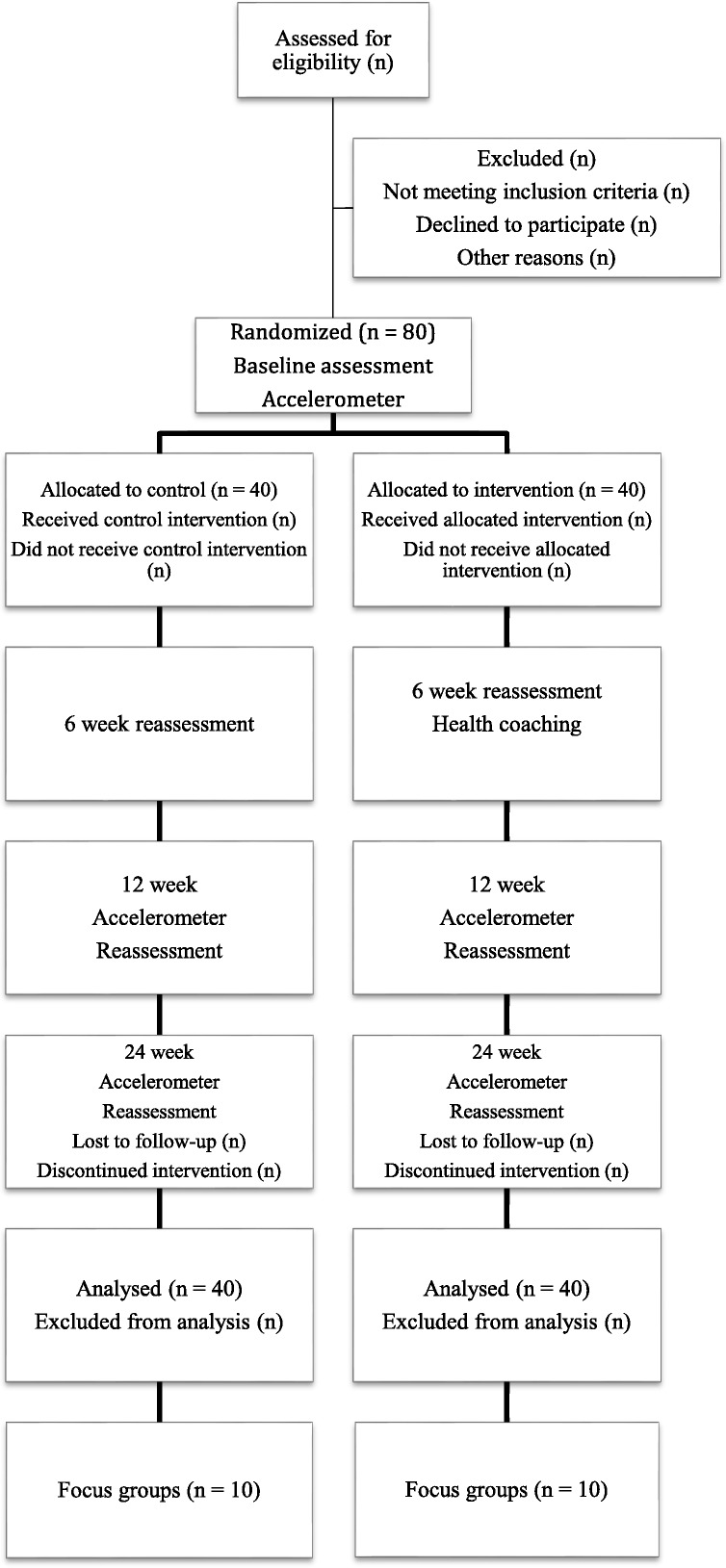
CONSORT 2010 Flow Diagram for SMART-MOVE study

This study is a prospective single-blinded randomized controlled trial (RCT) for a period of 6 months. Participants will be randomized 1:1 between the intervention and standard care. The data analysed will be based on intention-to-treat analysis. After receiving informed consent from the participants, baseline measures will be collected. Figure 1 illustrates the study design.

### Recruitment

Participants recruited are community-dwelling older people who present to TQEH through the Emergency Department, Acute Medical Unit, Geriatric Evaluation and Management Unit and Geriatrics Medicine Outpatients (including clinics at the Adelaide Geriatrics Training and Research with Aged Care [G-TRAC] Centre). Potential participants are approached by treating clinicians, and if these participants express interest, further contact will be made by one of the research team members to explain the study. Participants will also be recruited from community-based falls prevention programs (i.e. Resthaven Incorporated, Southern Cross). Research personnel will attend the programs and contact telephone number and e-mail will be provided for participants to express their interest. Flyers will be distributed through established community-based organisations for seniors such as Council for the Ageing. This study will also be advertised in newsletters of these organisations. Community talks about sedentary behaviour and falls at various seminars will be provided by the research personnel. Those who attended are provided with a contact telephone number and e-mail to express their interest.

### Participants

Participants will be screened as positive if they are aged over 65 years with a rapid cognitive screen of six and above [36] and scores 4 or higher on the STEADI falls risk self-assessment tool [29]. The STEADI toolkit is a broad, evidence-based resource validated to assess falls risk and individualize fall interventions into clinical practice.

Participants are included if they are screened positive, community-dwelling, can walk independently up to 10 m with or without walking aid, are conversant in English and have scores of 1 or more on the FRAIL screen [37].

Participants are excluded if they are unable to participate in the proposed intervention, have moderate to severe dementia, unable to walk independently up to 10 m with or without walking aid, are in the terminal phase of illness, plans to move out of the metropolitan area within 6 months of the screening clinic visit, plan to be away for more than 2 consecutive weeks during the study intervention period or are participating in another similar physical activity interventional study or programme. There is no specific exclusion criterion in relation to current physical activity levels.

Participants will be screened for eligibility by research personnel and provided with an information pack. The initial screening occurs over a telephone interview. If potential participants are interested, they will inform one of the researchers via telephone or email. Those who express interest will be contacted by a research personnel to have the study described further and their willingness to participate determined. They will be given approximately a week to decide on their participation. If they express their intention to participate, they will attend the study centre (either TQEH or Adelaide G-TRAC Centre) for consent and baseline measurements. When possible, the reasons for declining participation will be recorded. Participants will attend the health centre by their own arranged transport.

### Consent

All participants will provide written consent. Participants will be advised of their right to decline participation and to withdraw at any time without giving any reasons. If they withdraw, research team members will not collect additional personal information from them after the time of their withdrawal. Participants will inform the researchers if they want their personal and clinical information already collected to be discarded or if consent is provided, information already collected be retained and analysed for the study. This will be possible as data will only be analysed after completion of the study.

### Randomisation and blinding

Randomisation and allocation of a participating group to intervention will take place after each participant have provided their consent and completed the baseline assessment. To ensure allocation concealment, the allocation will be determined after baseline data collection using a sealed envelope technique. The randomisation sequence will be computer-generated (https://www.randomizer.org/). We will stratify randomisation by sex, to allow equal numbers of men and women in each group. The research assistants in charge of collecting and entering all outcome data will be blinded to intervention allocation throughout the trial. Participants will be informed not to reveal their group allocation to the research assistants performing the assessments. Additionally, research assistants will be informed not to ask participants which group they are allocated to. Research assistants will be trained to ensure consistency with data collection. The number of participants in whom the research assistants are inadvertently unblinded at follow-up will be recorded.

### Intervention group

The intervention has been reported according to the Template for Intervention Description and Replication (TIDieR) (Table 1) [35]. The intervention group will be:

(a) Presented with the objective information regarding their sedentary levels based on the accelerometer recordings (mean daily sedentary time and step counts, and the sedentary time as a percentage of waking hours, measured over 1 week);

(b) Educated about the benefits of exercise by providing them with written information (i.e. the Choose Health: Be Active booklet);

(c) Set goals to decrease their sedentary time by using two face-to-face health coaching sessions, followed by four fortnightly phone calls; and

(d) Using a pedometer for daily step counts as a motivational tool. Pedometer readings will be entered into a diary. Participants will calculate the mean daily steps over 7 days (as shown by research personnel) and in the following week, participants are encouraged to increase their average daily steps by 200 as a goal.

The face-to-face coaching will occur at weeks 0 and 6 (T0 and T6) of the study.

Table 2 describes the content of the health coaching sessions classified according to the CALO-RE taxonomy of behaviour change techniques [28, 38]. All of the techniques will be used during the face-to-face coaching at week 0 and 6. Review of goals, identification of barriers with problem-solving, use of follow-up prompts and relapse prevention will be discussed during telephone interviews at week 2,4, 8 and 10. Goals will be set according to the SMART (Specific, Measurable, Attainable, Realistic and Time-bound) principles. Each participant will choose six ways to decrease their sedentary time and break up prolonged sitting or lying time from a list of pre-specified behavioural items combined with suggestions of their own (Table 3). For example, the first week, a participant may set a goal of breaking up his reading time every 30 min and then in the third week, this participant will add another goal such as walking once a day in the nearby park. Each goal is intended to increase the upright time gradually. The aim is for one goal to be integrated incrementally every fortnight, so that in the final fortnight, participants would have integrated six goals into their daily routine to reduce sedentary time. Activities that participants are encouraged to engage will involve them being in an upright position instead of sitting or lying. In partnership with the investigator, this tailored intervention will attempt to overcome some of the perceived barriers. Participants will be provided with a diary to record if they attempted or achieved their goals set for that period of time. The diary will be collected at the end of the study.

**Table 2:** Content for intervention by session based on the CALO-RE taxonomy of behaviour change techniques

Behaviour change technique	Session 1	Session 2	Session 3	Session 4	Session 5	Session 6
	In person	Telephone	Telephone	In person	Telephone	Telephone
Shaping knowledge about the health consequences of sedentary behaviour	X			X		
Goal-setting (behaviour)	X			X		
Goal-setting (outcome)	X			X		
Feedback on behaviour	X			X		
Action planning	X	X	X	X	X	X
Barrier identification/problem solving	X	X	X	X	X	X
Use of follow-up prompts	X	X	X	X	X	X
Review of goals				X		
Relapse prevention/coping planning		X	X	X	X	X

*CALO-RE* Coventry, Aberdeen and London—Refined

**Table 3:** Domains for reduction of sedentary time

Domestic—indoors	Watching television or videos
Domestic—outdoors (e.g., gardening)	Screen-based activities (e.g. computer or tablet)
Transportation	Reading
Recreation	Others

A health coach with medical or nursing background will maintain contact through telephone calls, with participants in the interventional group to monitor progress towards reducing sedentary behaviour and to assist participants to overcome any participation barriers that arise. During the fortnightly telephone contacts, health coaches will also enquire about the circumstance of any falls that participants may have experienced and they will discuss strategies for reducing the risk of future falls. Health coaches will undergo training using written materials prepared by the research team.

A pedometer enhanced with a web-interface, Fitbit Zip™ (San Francisco, CA, USA) will be provided to all intervention group participants to provide feedback on the number of steps achieved daily. These pedometers will be provided as a motivational tool to encourage a reduction in sedentary behaviour. Participants will be encouraged to wear the pedometer during waking hours on a daily basis for the whole 12-week intervention period to record their daily steps and provide feedback and motivation to reduce their sedentary behaviour. Participants will be able to keep the pedometer at the completion of the study.

Participants in the intervention group will also be encouraged to attend falls prevention classes provided through one of the local allied health facilities (http://fallssa.com.au/). The aim of these classes is to help address some of the participants’ risk factors for falls. The participants will pay a small fee to attend these classes. The community aged care service providers will offer these classes. If participants have attended one of these classes in a 12-month period prior to enrolment, they will not be required to repeat these classes. They will be provided with information about what participant should do in the event of a fall (Standing Up to Falls—SA Health).

Goal attainment scale (GAS) is a method for quantifying progress towards defined goals [39, 40]. Three goals to reduce sedentary behaviour will be established at baseline by participants in the interventional group using the GAS with assistance from the health coach. Another three goals will be determined at the end of week 6. Once the goals are agreed upon, the health coach and participant will then predict the GAS outcomes on a five-point scale ranging from − 2 to + 2, where a score of 0 indicates achievement of the set goal, a score of − 1 indicates no change from the baseline level of ability for that goal type, − 2 indicates worse performance than at baseline and + 1 and + 2 indicate ‘somewhat better’ and ‘much better’ performance than the set goal, respectively. Attainment of the agreed goals to reduce sedentary behaviour will be assessed at both six and 12 weeks after participant randomization by a research assistant who is unaware of group assignment. The motivation for change will be evaluated using the Change Questionnaire [41]. Participants’ motivation will be assessed at T0, T6, T12 and T24.

### Control group

The control group will receive written information about the benefits of being active (i.e. the Choose Health: Be Active booklet) and will be provided with their accelerometer data readings during the one face-to-face meeting they have. Like the intervention group, they will be provided with information about what participant should do in the event of a fall. They will also be encouraged to join a falls prevention class if they have not participated in one in the preceding 12 months. No goals will be set and they will not receive any fortnightly phone calls to minimize any confounding motivational effects that might occur. Therefore, the only addition over and above usual care would be the provision of their baseline status from the first accelerometer assessment.

### Primary outcomes

#### Feasibility

Feasibility indicators include the trial process (recruitment, retention, acceptability, adherence and safety) in the study. Adherence to the intervention will be assessed according to completion of the goals set and fidelity to phone calls and visits. Acceptability of the intervention will be assessed with a questionnaire.

#### Sedentary time

Total sedentary time will be measured by ActivPAL™ (PAL Technologies Ltd., Glasgow, UK), a small (35 × 53 × 7 mm, 15 g) uniaxial accelerometer-based device attached anteriorly on the right mid-thigh held in place by a waterproof dressing (3 M Tegaderm transparent dressing) for 24 h over 7 days. This device uses accelerometer-derived information about thigh position to estimate time spent in different body positions (i.e sitting or lying, standing and stepping). Data will be collected for a 1-week period and processed in 15-s epochs using the ActivPAL™ software (version 5.8.3). The ActivPAL™ has been tested for reliability and validity, has been used with older adults and is considered the current most valid objective measure of sedentary behaviour [20]. The duration spent sitting, standing, lying and moving daily will be quantified.

### Secondary outcomes

Secondary outcomes measured include self-reported sedentary time, FES, change in gait speed and proportion of fallers and numbers of falls. Self-reported sedentary time will be evaluated using the validated MOST questionnaire [30]. Fear of falling will be assessed using the self-reported short-form FES International [31]. Gait speed will be assessed during a 6-m walk. The number of falls in each participant will be assessed during the 6 months of the study by self-report. Explanation will be provided to participants that a fall is defined as an event during which a person inadvertently comes to rest on the ground, floor or other lower level [42].

### Assessments

Assessment of outcome measures will occur at three time points—baseline (T0), week 12 (T12) and week 24 (T24). Assessments performed at these time points are summarized in Table 4.

**Table 4:** Methods and timing of assessing variables in this study

Variables	Measure	When	Time to complete
BMI (weight and height)	Calibrated digital scales and stadiometer	T0, T12, T24	1 min
Cognition	Rapid Cognitive Screen	T0	5 min
Cognition	Trail Making Test	T0, T12, T24	8 min
Mood	Geriatric Depression Screen-Five Item	T0	2 min
Frailty	FRAIL screen	T0	2 min
Personality	10-item personality inventory	T0	3 min
Self-reported physical activity	IPAQ—Elderly	T0, T12, T24	5 min
Self-reported sedentary behaviour	MOST questionnaire	T0, T12, T24	5 min
Social Engagement	Lubben Social Network	T0	2 min
Review of falls episode	History-taking	T0, T6, T12, T24	5 min
Fear of falling	Falls efficacy scale	T0, T12, T24	4 min
Activities of daily living	Katz ADL	T0, T12, T24	5 min
Quality of life	EQ5D	T0, T12, T24	2 min
Nutritional status	Mini-nutritional assessment—short-form	T0, T12, T24	2 min
Gait speed	SPPB	T0, T12, T24	2 min
Grip strength	SPPB	T0, T12, T24	5 min
Balance test	Berg Balance	T0, T12, T24	15–20 min
Appendicular lean muscle mass	Bioelectrical impedance	T0, T12, T24	5 min
Motivation to change	Change Questionnaire	T0, T6, T12, T24	5 min
Goal attainment	Goal Attainment Scale	T0, T6, T12, T24	3–6 min
Sedentary time	Accelerometer	T0, T12, T24	
Step counts	Pedometer	T6, T12, T24	2 min
Pedometer adherence	Pedometer	T6, T12, T24	5 min
Neighbourhood Environment	NEWS	T0	15 min

*ADL* activities of daily living, *BMI* body mass index, *IPAQ* International Physical Activity Questionnaire, *MOST* Measure of Older Adults’ Sedentary Time, *NEWS* Neighbourhood Environment Walkability Scale, *SPPB* Short Performance Physical Battery

Demographic information will be collected at T0. This information includes date of birth (age), ethnicity, living arrangement, comorbid conditions and medication use. The Charlson comorbidity index (CCI) will be applied to measure the burden of disease and thus enable confounder adjustment of the results [43]. The index is based on 22 clinical conditions. Each condition is given an associated weight (1, 2, 3 and 6) according to the gravity of the disease.

Anthropometrics include height (m), weight (kg) and body mass index (BMI) (kg/m^2^). To ensure accurate measurement, height will be measured to the nearest 0.01 unit and weight to the nearest 0.1 unit. Participants will be instructed to remove their shoes and any bulky clothing before measurement. Height will be measured using a portable stadiometer, and weight will be measured using a calibrated electronic scale.

A ten-item personality inventory will be completed by the participants at baseline to determine their Big-Five personality domains [44]. Self-report of the ability to perform personal activities of daily living (ADLs) were assessed using the Katz ADLs [45]. The Luben Social Network Scale will be used to assess the level of social engagement [46]. Generic physical activity and sedentary behaviour will be self-reported using the elderly version of the International Physical Activity Questionnaire (IPAQ) [47] and a validated sedentary behaviour questionnaire measuring older adults’ sedentary time (MOST) [30]. Participants will also complete the Neighbourhood Environment Walkability Scale (NEWS). This scale will assess participants’ perception of neighbourhood design features related to physical activity, including residential density, land use mix (including both indices of proximity and accessibility), street connectivity, infrastructure for walking/cycling, neighbourhood aesthetics, traffic and crime safety and neighbourhood satisfaction [48]. The European Quality of Life (EQ5D) scale will be used to estimate the health status and quality of life [49]. Mood will be assessed using the Geriatric Depression Scale—five-items [50].

Physical function will be assessed objectively using the short physical performance battery [51]. Total functional capacity is based on a composite score from the following subtests: repeated chair stands, balance (semi-tandem, side-by-side stand, tandem stand) and 6 m walk to assess gait speed. Bergs Balance Scale will be used to measure balance [52]. Grip strength will be measured using the Jamar hand dynamometer (Model J00105 JAMAR Hydraulic Hand Dynamometer, Sammons Preston, Bolingbrook, IL, USA) with the dominant hand in a seated position with their feet firmly on the ground and back straight against the chair (to prevent participant movement) [53]. Three trials will be made with a pause of about 10 to 20 s between each trial to avoid the effects of muscle fatigue. Maximum grip strength is then recorded.

The Trail Making Test is a neuropsychological test of visual attention and task switching. It consists of two parts in which the subject is instructed to connect a set of 25 dots as quickly as possible while still maintaining accuracy. The test can provide information about visual search speed, scanning, speed of processing, mental flexibility, as well as executive functioning [54]. It is sensitive to detecting cognitive impairment associated with dementia [55]. Fear of falling will be assessed using the self-reported short-form FES International [31].

Appendicular skeletal muscle mass (ASM) will be assessed using the single frequency Quantum II Body Composition Analyzer (RJL Systems, Clinton Township, MI, USA). This device produces a 425 μA constant sinusoidal current at a single frequency of 50 kHz ± 1%. Participants will be asked to remove their shoes, socks and jewelry. Bioelectrical impedance analysis (BIA) measurements are performed with participants in a supine position, with their arms positioned by their sides and palms facing down on a non-conductive examination chair. Two electrodes are placed on the right wrist and two others on the right ankle. On the wrist, one electrode is placed on the dorsal aspect next to the ulnar head and another is placed on the dorsal surface of the first joint of the middle finger. On the ankle, one electrode is placed on an imaginary line bisecting the medial malleolus and the other electrode on the base of the second toe. The device reports resistance and reactance values. ASM will be estimated using the prediction equation by Sergi et al [ASM (kg) = − 3.964 + (0.227 × RI) + (0.095 × weight) + (1.384 × sex (men = 1, women = 0) + (0.064 × Xc) where RI: resistance index, Xc: reactance] [56].

### Focus groups

We will conduct four focus groups with participants from the intervention (two groups) and control (two groups) arms at the end of the intervention at week 24. These sessions will consist of groups with four to eight individuals and last for 1.0 to 1.5 h. A trained professional will facilitate these focus groups, with the assistance of a second researcher. The focus groups will evaluate feasibility, including acceptability, and evaluate how the accelerometer and pedometer affected the participants and their behaviours. For the intervention group, the perceived effectiveness of the SMART-MOVE health coaching will also be explored. With consent, focus groups will be audio recorded and transcribed. Thematic analysis will be conducted, focusing on analysing the data to answer the research questions as well as using an inductive approach.

### Statistical analyses

Feasibility findings will be primarily descriptive and used as a metric for improvement when compared to similar studies. We hypothesize that the intervention will be feasible among several improvement metrics including 40% or more recruitment rate and less than 20% attrition. This hypothesis is based on findings from earlier studies [13–16].

Descriptive analyses will be conducted using mean with standard deviation, median with interquartile range and frequency for baseline characteristics. These characteristics include age, gender, ethnicity, physical function, health status, quality of life and mental status. Mean group differences will be examined using independent samples *t* test for normally distributed data and nonparametric tests (Kruskal-Wallis and Mann-Whitney *U*) for non-normally distributed data. Differences in frequency of variables will be compared using chi-square test.

Analysis of variance (ANOVA) will be used to test the post-intervention difference at 12 and 24 weeks between the two groups. The covariates in the analysis will include baseline values of the dependent variable and any variable significantly different between groups at baseline. Group mean values for adherence variable at 12 and 24 weeks (retention rate, days the accelerometer and pedometer are worn) will be analysed by an independent *t* test. The data analysed will be based on the intention-to-treat analysis.

### Power calculation

Sixty-four participants will be required to detect a 30 min/day reduction [15] in sedentary time (80% power, alpha 0.05) in the interventional group, assuming there is no change in the control arm. Therefore, the aim is to recruit 80 participants (40 in each arm) to allow for 20% attrition. The same number of male and female participants will be recruited.

## Discussion

This study is highly important because of the dual significance of falls and sedentary behaviour among older people. This trial is the first to integrate strategies using feedback from accelerometer and goal-setting health coaching in older people who have had a fall in the last 12 months or are at risk of falling.

Currently, there is limited work on community-based interventions to reduce sedentary behaviour in older people that can alter the risk of falls. This trial will address a key gap in the current evidence regarding effective ways to reduce sedentary behaviour in older people and the use of an accelerometer as a tool to motivate change in behaviour. It will provide a model for integrated falls and sedentary behaviour assessment and reduction programme that could be implemented in community health care settings. If such intervention with health coaching on sedentary behaviour is effective in reducing falls, it has enormous potential benefit for older people and the health care system. The trial findings will be disseminated in peer-reviewed journals and through scientific and professional conferences. The target in step count of an average of 200 steps per week increment was adopted for this study because it was seen as a realistic increase to achieve in 1 week. One previous study has used 1500 steps over 4 weeks which would be an average of 375 steps/week increment [57]. This has been revised to a lower target given that our participants are at risk of falls and may require a more gradual increment. Since no evidence exists to guide goals for sedentary time, behaviour change researchers suggested a 30-min improvement in sedentary time would be realistic for participants to achieve during the 12-week intervention [13, 57].

One of the limitations of the study is that the research personnel performing the assessment may not be fully blinded to the group participants that are allocated into because participants may inadvertently reveal this information. In addition, this is a small study and is not adequately powered to evaluate the effects of this intervention on the rate of falls. The duration of the study is also relatively short. Therefore, the sustainability of any behaviour changes beyond the study period is uncertain. Information about any falls in the control group is collected over a 6-week period which can lead to inaccurate recall from participants. However, it serves as a useful pilot study that can be used to enhance future health coaching programs on reducing sedentary behaviour among older people at risk of falls.

## Abbreviations

ADLs: Activities of daily living
ANOVA: Analysis of variance
ASM: Appendicular skeletal muscle mass
BIA: Bioelectrical impedance analysis
BMI: Body mass index
CCI: Charlson comorbidity index
CONSORT: CONsolidated Standards of Reporting Trials
EQ5D: European Quality of Life
FES: Falls efficacy scale
GAS: Goal Attainment Scale
IPAQ: International Physical Activity Questionnaire
MOST: Measure of Older Adults' Sedentary Time
MVPA: Moderate to vigorous physical activity
NEWS: Neighbourhood Environment Walkability Scale
SDT: Self-determination theory
SMART: Specific, Measurable, Attainable, Realistic, Time-bound
SPIRIT: Standard Protocol Items: Recommendations for Interventional Trial
SPPB: Short physical performance battery
TIDieR: Template for Intervention Description and Replication
